# The Hospital Problem: The Future of the Funds

**Published:** 1911-05-06

**Authors:** 


### The Hospital

A JOURNAL OF

**The Medical Sciences and Hospital Administration.**

NEW SERIES. No. 222, VOL. IX. [No. 1294, VOL. L.] SATURDAY, MAY 6, 1911

### THE HOSPITAL PROBLEM: THE FUTURE OF THE FUNDS.

LAST week we briefly discussed the tendency which has been observed in some quarters to ascribe to the large hospital funds a disposition to assume the rôle of despotic trusts. We then expressed the opinion that the influence of the funds has been in the past, and is bound to be in the future, so long as the principles which underlie the voluntary system at its best are maintained, of the utmost benefit to hospitals in this country. Now it is necessary to devote some attention to a question which arises out of the consideration of the relation of these great funds to our hospital world generally, and that is: "What place will be assigned to these important collecting and controlling agencies in a scheme of which the basis is co-operation between State and voluntary hospital, between representatives of the ratepayers on the one and representatives of the generously charitable public on the other hand?"

In order to arrive at the answer to this question—which must, of course, be to a certain extent tentative, since the true reply to such an inquiry is only developed after many things have been tried and considered—it is helpful to bear in mind what has taken place in other countries. Here, once more, the German example is instructive. For a full description of the decline and fall of certain large voluntary collecting agencies which at one time existed in the German Empire, and which the establishment of a general non-coöperative system of State grants to all hospital institutions swamped and destroyed, we must refer readers to that interesting and instructive résumé by Dr. Grotjahn of the relations between the German hospitals and the State insurance system.* The history of the contributing funds in Germany shows that when State aid, in an unrestrained, uncoöperative manner, has been given to institutions for the sick and distressed, the amount of voluntary charitable help falls—a result which is only to be expected. When the public knows that beds are to be paid for out of the rates, and when the claims of every hospital, no matter what work it does and what results it shows, are reckoned equal, there is practically no inducement to progress and advance. The superintendent, harassed by the constant worries of official red-tape, finds it inexpedient in some, impossible in other cases, to devote the necessary sums to reconstruction and repair of buildings and equipment. On the other hand, where a system of grants in aid, calculated on the amount of free contributions made by the public to the institution, prevails, it appears to have materially stimulated the generosity of the public, and to have led to a gratifying increase in the voluntary subscriptions. The figures available for certain German districts where such a system, or a modification of it exists, are instructive. With the tendency, so prominent in Germany during the last decades of the nineteenth century, to make the State the supreme power in hospital control, the voluntary collecting and contributing funds, which were for the most part co-partnerships of working-men, the members of which contributed a certain fixed rate of money every quarter, have fallen on evil days. Only the best organised of these societies have survived, and have done so largely because they have taken care that the State aid should not work in a demoralising manner. A corollary to this principle, it is interesting to note, is the gradual separation of the funds from the State institutions. There is a remarkable tendency on the part of the voluntary contributing societies in Germany to establish their own hospitals. It is true this tendency cannot be ascribed to a single cause, but in a sense it is due to the dissatisfaction which is felt with the State management of the hospitals. Hospital workers in Germany regard this separatist tendency with keen regret, for it must inevitably lead to the establishment of a series of private institutions, which are uncontrolled by outside influences, and which owe account of their management and results only to the societies themselves. Such societies, there is reason to fear, will develop into real trusts—trusts in which the public has nothing to say, in which the Government, under the present state of the law, cannot interfere, and which may be likened to the average nursing home. Already it is urgently desirable to arrange some compromise between the funds and the State-aided institutions, and to seek a method whereby the hospitals can deal efficiently and thoroughly with the mass of patients sent by the societies—virtually convalescent or chronically ill patients—in some practical way.

That being the case in a country where State-aid has been tried for so many years, and where the faults of a one-sided system are apparent to everyone who takes the trouble to investigate conditions thoroughly, it is of the first importance to us in this country to see that in any system which is evolved out of the flux that prevails at present, due care is taken that the interests of the public are safeguarded. We already possess an admirable means of attaining that end in the existing great funds, of which the chief is the King's Fund. It will be necessary, however, to create branches of this fund in every large hospital town in the kingdom, and, for various reasons, to establish a central council or controlling body, thoroughly representative of all the various and different interests to be found among the contributors.

In a co-operative scheme such a central fund, on the basis of the King's Fund—in other words, an extension of that fund—will have a real and practical place of usefulness. Firstly, it will be an advisory body; secondly, it will be an expert controlling body, whose findings and recommendations every institution in the kingdom will have to respect, not because of any legislative enactment or direct coercion, but simply through the fact that they will represent the best and most authoritative criticism on hospital management and policy. It is needless to point out the vast utility of such an assisting body in a co-operative scheme between State and voluntary effort. By it the interests of the community will be effectively safeguarded, and the generosity of private charity directed into the best channels where it is most likely to be productive of the best results. A simple system of State or municipal control must inevitably mean the decline of the voluntary principle—a result which no one who is alive to the advantages which accrue from voluntary effort is likely to contemplate with equanimity. We have already stated that we are of opinion that the best way out of existing difficulties will be by the creation of a system of practical co-operation between the State, municipality, and the voluntary subscriber. We are convinced that it will be of material benefit to the community if such a scheme has the added assistance of the experience and influence of the councils of the great funds. For the many years they have now been in existence these bodies have done work that it is impossible to rate too highly, and it would be foolish and absurd to relegate them to the background when a new scheme is instituted. The funds have come to stay, and in any new system that is to be evolved they should be reckoned with, and due regard should be paid to their claims—claims which every hospital worker and every member of the public who is acquainted with the work that has been done by the great funds will readily acknowledge.

*** Krankenhauswesen und Heilstattenbewegung Im Lichte der Scialen Hygiene. Von Dr. Med Alfred Grotjahn.

